# Manganese Exposure Is Associated with Reduced Grip Strength: Evidence from Humans and Mice

**DOI:** 10.3390/toxics14010031

**Published:** 2025-12-26

**Authors:** Peiyu Fang, Chuanqiao Tang, Shengtao Wei, Wenmin Lu, Shaohui Liu, Xiaoli Ma, Li’e Zhang, Guiqiang Liang, Jian Wang, Yang Peng, Yunfeng Zou

**Affiliations:** 1 Department of Toxicology, School of Public Health, Guangxi Medical University, Nanning 530021, China; 2 Guangxi Key Laboratory of Environment and Health Research, Guangxi Medical University, Nanning 530021, China; 3 Department of Occupational and Environmental Health, School of Public Health, Guangxi Medical University, Nanning 530021, China

**Keywords:** manganese, grip strength, elderly men, C57BL/6J mice, inflammatory cytokines

## Abstract

Although excessive manganese (Mn) exposure is known to cause neuromotor function in cases of poisoning, its effect on grip strength (a neuromotor marker) in older adults at environmental levels remains unclear. To investigate this issue, we conducted an integrated investigation combining epidemiology and animal experimentation to examine the association between urinary manganese and grip strength. A cross-sectional study of 375 elderly men (60–74 years) was conducted in Guangxi, China, from 2016 to 2017. Urinary Mn concentrations were determined by ICP-MS, and their associations with grip strength were evaluated using generalized linear models and restricted cubic splines. In parallel, 32 six-week-old male C57BL/6J mice were exposed to 0, 5, 10, or 15 mg/kg MnCl_2_·4H_2_O via intraperitoneal injection for 6 weeks. Forelimb grip strength of the mice was measured after the final exposure, and mRNA expression of inflammatory markers and cytokines (C reactive protein (CRP), interleukin (IL)-6, and tumor necrosis factor (TNF)-α in triceps) in triceps tissue was quantified. The median urinary Mn concentration in the study population was 0.22 μg/g creatinine. After adjusting for confounders, urinary Mn was inversely associated with hand grip strength (highest vs. lowest tertile: β = −3.57 kg; 95% CI: −5.68 to −1.47; *p*-trend = 0.007). Similarly, in male C57BL/6J mice, grip strengths declined significantly with increasing Mn exposure (*p*-trend < 0.0001), accompanied by upregulation of the mRNA levels of CRP, IL-6 and TNF-α in muscle tissue. Together, our findings suggest that environmental manganese exposure is inversely associated with grip strength in elderly men. While the manganese doses used in the animal study exceeded typical human environmental exposure, the experimental results further indicate that such grip strength reduction may be linked to muscle inflammation.

## 1. Introduction

Manganese (Mn) is an essential trace element that may also exert toxic effects upon excessive exposure. This dual nature has drawn significant attention in environmental health research. While daily dietary intake generally satisfies physiological manganese requirements, growing environmental contamination—driven by human activities such as mining, battery manufacturing, and the use of methylcyclopentadienyl manganese tricarbonyl (MMT) Fern (MMT) [1] has increased the risk of overexposure in the general population. Guangxi, China, endowed with the most abundant manganese ore resources, has particularly prominent issues regarding manganese exposure in both its natural environment and population [2,3], underscoring an urgent need for systematic research.

The adverse health effects of excessive manganese exposure, particularly on the nervous system, have been increasingly documented. These include memory impairment [4], intellectual disability [5,6], and motor dysfunction [7]. Among motor abnormalities, diminished hand coordination stands out as a typical manifestation, implying a potential direct effect of manganese on hand muscle function [8,9,10]. However, evidence regarding the association between manganese exposure and grip strength, a core indicator of hand muscle strength, remains limited and inconsistent. Only three epidemiological studies have addressed this issue, yielding conflicting conclusions: a cross-sectional study in Guangxi, China, reported an inverse correlation between urinary manganese levels and grip strength [11], whereas two analyses of U.S. NHANES data found no such association [12,13]. In vivo studies examining manganese’s effect on grip strength in animal models have also produced inconsistent results [14,15,16]. These discrepancies highlight the need for more in-depth research to clarify the relationship between manganese exposure and grip strength.

As a representative measure of overall muscle function, grip strength is widely utilized in clinical and epidemiological settings due to its simplicity, cost-effectiveness, and non-invasiveness [17]. Beyond reflecting muscular condition, it is also regarded as a biomarker of aging [18], typically peaking in early adulthood and gradually declining thereafter [19]. In older adults, reduced grip strength is associated with elevated risks of cardiovascular disease [20,21], neurodegenerative disorders [22,23], general morbidity [24] and mortality [24,25]. Against the backdrop of a rapidly aging global population, identifying modifiable risk factors for grip strength decline is critical for promoting healthy aging. Emerging evidence suggests that exposure to environmental pollutants, such as air pollutants [26], phthalates [27], and heavy metals (e.g., cadmium (Cd)) [28,29], may contribute to diminished grip strength in the elderly. Nevertheless, the role of manganese, a prevalent heavy metal with substantial exposure risk, remains poorly understood and warrants further clarification.

Mechanistically, excessive Mn exposure may induce toxicity through inflammatory pathways, as evidenced by respiratory symptoms [30], neuroinflammation [31,32], and elevated systemic levels of inflammatory cytokines [33]. Furthermore, a recent meta-analysis indicated that higher circulating concentrations of inflammatory markers—including C reactive protein (CRP), interleukin (IL)-6, and tumor necrosis factor (TNF)-α are associated with reduced hand grip strength in adults [34]. These findings suggest that inflammatory mechanisms may mediate the relationship between Mn exposure and decline in grip strength; however, this hypothesis has not yet been empirically verified. Thus, we sought to investigate whether CRP, IL-6 and TNF-α are involved in the potential effects of Mn exposure on grip strength.

Based on these research gaps, we hypothesized that manganese exposure is associated with reduced grip strength in the elderly and that this association may be mediated by CRP, IL-6, and TNF-α. To test our hypothesis, we first examined the relationship between urinary Mn exposure levels and hand grip strength in a cross-sectional study of 375 elderly men. Additionally, we established a mouse model to evaluate the impact of Mn exposure on forelimb grip strength and the expression of inflammatory markers (CRP) and cytokines (IL-6, TNF-α) in muscle tissue.

## 2. Materials and Methods

### 2.1. Epidemiological Study

#### 2.1.1. Study Population

A cross-sectional study was carried out from August 2016 to July 2017 in a rural area of Hechi city, Guangxi Zhuang Autonomous Region, China. A total of 375 elderly men who fulfilled the following criteria were recruited: (1) residents in the study area; (2) ≥60 years old; (3) individuals who agreed to receive a questionnaire survey and provide urine samples; (4) individuals who did not suffer from severe diseases and had the ability to communicate with the interviewers. Other details of the survey have been described previously [35,36]. All human participants included in this study had fully understood the research purpose, process and potential risks before participating in the study, and all signed written informed consent forms in person. This study protocol received ethical approval (Approval No. 20140305-022) from the Medical Ethical Committee of Guangxi Medical University. The research was conducted in accordance with the principles of the Declaration of Helsinki and complied with China’s “Ethics Review Measures for Biomedical Research Involving Humans”.

#### 2.1.2. Urinary Mn Measurement

We used polypropylene tubes to collect morning urine samples and stored them at −80 °C before further analyses. Urinary Mn as well as Cd concentrations were measured using an inductively coupled plasma mass spectrometer (ICP-MS; NexION 350X, PerkinElmer Inc. Shelton, CT, USA). The protocol of urinary metals measurement had been described in our previous works [35,36]. Briefly, urine samples were thawed at room temperature (22 ± 2 °C) and mixed adequately using a vortex oscillator for 30 s, then 1 mL of urine sample and 5 μL of 65% *v*/*v* HNO_3_ (Merck, Darmstadt, Germany) were added to the same polypropylene tube for overnight nitrification at 4 °C, after which 9.0 mL of 1% *v*/*v* HNO_3_ was added to dilute the samples to 10 mL the next morning. The samples were further sonicated by ultrasound at 40 °C for 1 h, and equilibrated to room temperature before detection. For quality control, Standard Reference Material (SRM2670a Toxic Elements in Urine; National Institute of Standards and Technology, Gaithersburg, MD, USA) was used between every 20 samples to assess the instrument performance, and the concentrations measured were within the ranges provided. The low and high concentration recoveries of Mn and Cd were 94.03%, 93.12% and 101.13%, 100.20%, respectively. The limit of detection (LOD) values for Mn and Cd were 0.047 μg/L and 0.012 μg/L, respectively, with detection rates being 94.93% and 100.00%, respectively. Urinary Mn concentrations below LOD were replaced by half of the LOD.

#### 2.1.3. Hand Grip Strength

Dominant hand grip strength was measured using a WCS-100 Digital Grip Strength Dynamometer (Shanghai, China). Participants were told to stand relaxed with feet shoulder-width apart and arms dropped naturally, to squeeze the dynamometer as hard as possible using their dominant hand, and to avoid bending their arms, bending down, or letting the dynamometer touch their bodies. The record (kg) was read when the reading was stable, with reading accurate to one decimal place.

#### 2.1.4. Covariates

Baseline characteristics of the participants, including age, ethnicity, marital status, occupation, household income, education level, tobacco smoking status, alcohol drinking status, and farm-work doing status, were collected using standardized and structured questionnaires in the face-to-face interview. Weight and height were measured in standardized conditions, and body mass index (BMI) was calculated as weight divided by height squared (kg/m^2^). Blood pressure was measured using an electronic sphygmomanometer and hypertension was defined as a history of taking antihypertensive medicines, or a systolic blood pressure of at least 140 mm Hg, or a diastolic blood pressure of at least 90 mm Hg, according to the international standard [37].

### 2.2. Animal Study

#### 2.2.1. Animals and Treatment

Thirty-two five-week-old male C57BL/6J mice (specific pathogen free) were purchased from GemPharmatech company (GemPharmatech Co., Ltd., Nanjing, China). Mice were housed in the Laboratory Animal Center of Guangxi Medical University under a 12 h light/dark cycle at 22–24 °C with free access to sterile food (KEAO XIELIFEED CO., Ltd., Tianjin, China) and water. All animals were acclimated for 1 week before experiments. Then, mice were randomly assigned to four groups (*n* = 8 per group): control (vehicle), 5, 10, or 15 mg/kg (Actual manganese content) MnCl_2_·4H_2_O. Mice were intraperitoneally (i.p.) injected with vehicle (physiological saline) or corresponding doses of Mn for 6 weeks (once a day and 5 times per week). Body weight was measured every 3 days. All animal experiments were conducted in accordance with the National Standard GB/T 35892-2018 [38]. The procedures involving mice were approved by the Animal Ethics Committee of Guangxi Medical University (Approval number: 201601135).

#### 2.2.2. Forelimb Grip Strength

After the last dose delivery, forelimb grip strength of each mouse was measured using a grip strength meter (UniBioLab, Beijing, China) equipped with a digital sensor. A 140 mm long and 95 mm wide stainless steel metal grip plate was assembled to the digital sensor. The grip bar diameter was 3 mm and the grip bar interval was 10 mm. According to the manufacture’s instruction and references [39], the mouse was held by the tail so that only the forelimb paws were grasping the grip bar, then the mouse was pulled back gently and steadily until its forelimbs were released. Each mouse was tested five times and the grip strength peak values (g) were recorded. The five recordings were averaged and standardized with the body weight (g) of the same day to obtain a final reading of forelimb grip strength (g/g of body weight).

#### 2.2.3. Triceps Isolation and Mn Determination

Mice were euthanized with avertin (0.5–0.6 mL per mouse) following the approval of the ethics committee, and three mice of each group were randomly chosen for perfusion fixation, thus the amounts of blood and tissue samples in each group were both five in the subsequent experiments. Mice forelimb skeletal muscle triceps were chosen to represent the forelimb region [40]. After being dissected, triceps tissues were quickly snap frozen in dry ice in 2 mL centrifuge tubes, and were then stored at −80 °C before further analyses. Triceps Mn concentrations were determined by ICP-MS. A part of wet triceps tissues was cut and weighed in order to standardize the Mn concentrations, then the triceps tissues were digested in 4.0 mL of *v*/*v* HNO_3_ using a high-throughput closed microwave digestion instrument (CEM, Matthews, NC, USA) complying with the following procedures: (1) climbing to 120 °C for 15 min and staying at 120 °C for 5 min; (2) climbing to 180 °C for 12 min and staying at 180 °C for 10 min; (3) climbing to 160 °C for 10 min and staying at 160 °C for 10 min; and (4) cooling down to <112 °C for 15 min. Thereafter, samples were heated and evaporated at 180 °C for 30 to 50 min until the liquid reached approximately 0.3 mL. Each sample was then diluted to 10 mL with deionized water and further sonicated by ultrasound at 40 °C for 1 h before detection. For quality control, Standard Reference Materials (Seronorm™ Trace Elements Urine L-1 RUO and L2 RUO, purchased from Sero™, Oslo, Norway; SRM 1640a, purchased from National Institute of Standards and Technology, Gaithersburg, MD, USA) were used, and the concentrations measured were within the ranges provided. The low and high recoveries of Mn were 96.21% and 95.48%, respectively. The Mn concentrations in triceps tissues (μg/g) were reported as a ratio of the Mn level to the wet weight of triceps.

#### 2.2.4. qRT-PCR Assay

Total RNA was extracted from mice triceps tissues using TRIzol Reagent (Invitrogen Co., Carlsbad, CA, USA) and reverse-transcribed to cDNA using a PrimeScript™ RT reagent Kit with gDNA Eraser (Takara Biotechnology Co., Ltd., Dalian, China) according to the manufacturers’ instructions. QRT-PCR was performed in duplicate with 2 μL cDNA per well using TB Green^®^ Premix Ex Taq ™ (Takara Biotechnology Co., Ltd., Dalian, China) on a StepOne Plus real-time PCR system (Thermo Fisher, Waltham, MA, USA) with the following procedures provided by the manufacturer: (1) denaturation at 95 °C for 30 s; (2) amplification at 95 °C for 5 s and 60 °C for 30 s for 40 cycles of PCR reactions; (3) and a melting curve analysis at 95 °C for 15 s, 60 °C for 60 s, and 95 °C for 15 s. Threshold cycle (Ct) values in duplicate were averaged, and the relative mRNA expressions of CRP, IL-6 and TNF-α were determined using the 2^−ΔΔCT^ method with GAPDH as the reference gene. The forward and reverse primer sequences are presented in Table S1.

### 2.3. Statistical Analyses

In the epidemiological study, descriptive statistics were conducted for participants’ baseline characteristics, with data presented as mean ± standard deviation (SD) or median (interquartile ranges) for continuous variables, and frequencies (proportions) for categorical variables. The association between urinary Mn concentrations and hand grip strength was explored using a general linear model. In this model, urinary Mn concentrations was included as a categorical variable (tertiles), and hand grip strength was included as a continuous variable. The nonlinear dose–response relationship between urinary Mn concentrations and hand grip strength was further explored using a restricted cubic spline (RCS) regression analysis with three knots fixed at 10th, 50th and 90th percentiles of urinary Mn concentrations distribution in all participants.

Confounding factors were included in the general linear model gradually. Model A was a crude model. Model B was adjusted for age, ethnicity, marital status, education level, and occupation. Model C was adjusted for model B covariates plus BMI, smoking status, drinking status, farm-work doing status, and hypertension, a chronic disease reported to be related with both urinary Mn concentrations [41] and hand grip strength [42]. Model D was adjusted for model C covariates plus tertiles of creatinine-corrected urinary Cd concentrations, because a recent study has reported that higher urinary Cd levels were associated with declined hand grip strength. Linear trend tests were conducted by including the median value of each tertile of creatinine-corrected urinary Mn concentrations as a continuous variable in the general linear models. The RCS regression model was adjusted for the covariates as in model D. Furthermore, general linear models were stratified by participants’ median age (66 years old), BMI subgroups (<18.5, 18.5–23.9, and ≥24.0 kg/m^2^), and tobacco smoking status (yes, no), and interaction terms were tested, in order to further evaluate the potential effects modified by age, BMI and smoking.

RCS regression analysis was performed using R software version 4.0.5 (The Comprehensive R Archive Network: http://cran.r-project.org (accessed on 15 October 2023)). Other statistical analyses were conducted with SAS version 9.4 (SAS Institute, Cary, NC, USA). Two-sided *p* < 0.05 was considered statistically significant.

In animal study, data were presented as mean ± SD unless otherwise noted. According to the data type, one-way ANOVA analysis or Kruskal–Wallis rank sum test was used to analyze the differences between the Mn-exposed and control groups. Mice bodyweight was analyzed by repeated measures ANOVA. Linear trend test for mice forelimb grip strength was conducted by including the median value of each group as a continuous variable in the general linear model. Statistical analyses were performed using IBM SPSS Statistics version 19.0. Graphical displays were performed using GraphPad Prism version 7.0. Two-sided *p* < 0.05 was considered statistically significant.

## 3. Results

### 3.1. Epidemiological Study

The baseline characteristics, creatinine-corrected urinary Mn concentrations, and hand grip strength are presented in Table 1. In the total 375 participants, the average age was 66.03 ± 3.92 years, and the average BMI was 21.69 ± 2.86 kg/m^2^. Most of the participants were Zhuang people (90.67%) and farmers (84.27%). Approximately half of the participants smoked or drank.

The median (interquartile ranges) value of creatinine-corrected urinary Mn concentrations was 0.22 (0.14, 0.42) μg/g creatinine, and the median (interquartile ranges) value of hand grip strength was 28.1 (22.3, 33.6) kg. Married individuals and non-farmers had lower urinary Mn levels (*p* < 0.05). Older individuals, those who accepted 1–6 years of education, and those whose BMI were lower than 18.5 kg/m^2^ had lower hand grip strengths (all *p* < 0.05).

**Figure 1:**
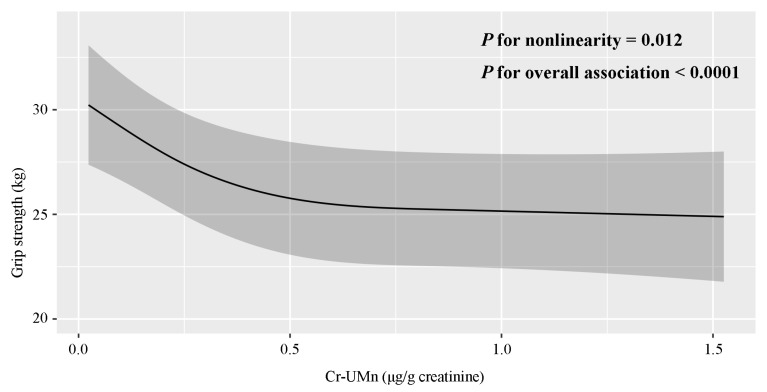
Restricted cubic spline regression model of urinary Mn levels and hand grip strength. The knots were set at 10th, 50th and 90th percentiles of creatinine-corrected urinary Mn concentrations distribution in all participants. The model was adjusted for age (60–64, 65–69, ≥70 years), ethnicity (Han, Zhuang, other), marital status (married, other), education level (no formal education, 1–6 years of education, >6 years of education), occupation (farmer, non-farmer), BMI (<18.5, 18.5–23.9, ≥24.0 kg/m^2^), smoking (yes, no), drinking (yes, no), farm-work (yes, no), hypertension (yes, no), and urinary Cd concentrations.

Table 2 illustrates the relationship between urinary Mn levels and hand grip strength. In the crude model (model A) and the models that adjusted for a series of confounding factors (model B, C and D), the results showed a negative association between urinary Mn levels and hand grip strength. In model D, participants in the highest tertile of urinary Mn had a 3.57 kg (95% CI: −5.68, −1.47) decrease in hand grip strength compared with those in the lowest tertile (*p*-trend = 0.007). The nonlinear relationship between urinary Mn levels and hand grip strength is further shown in Figure 1. The spline curve demonstrates a monotonic downward trend, and there was a dose–response relationship between creatinine-corrected urinary Mn levels and hand grip strength (*p* value for overall association was <0.0001). A major departure from linearity in the observed relationship was found (*p* value for nonlinearity = 0.012).

The results of the stratified analyses are shown in Table S2. The inverse association between urinary Mn levels and hand grip strength in elderly men remained more robust in individuals who were older (≥66 years), who had lower BMI (<24.0 kg/m^2^), and who were non-smokers (*p*-trend < 0.05), but the interaction term was only statistically significant in the smoking stratified analysis (*p*-interaction = 0.025).

### 3.2. Animal Study

**Figure 2:**
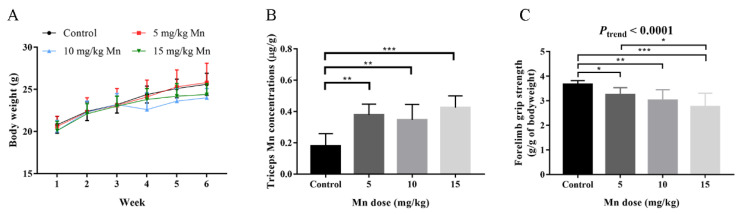
Effect of Mn exposure on mice bodyweight, forelimb grip strength, and triceps Mn levels. (**A**) The changes in bodyweight of control and Mn-exposed mice (*n* = 8 per group); data were presented as mean ± standard deviation. (**B**) Triceps Mn concentrations of control and Mn-exposed mice (*n* = 5 per group) determined by ICP-MS. (**C**) Forelimb grip strength of control and Mn-exposed mice (*n* = 8 per group) measured after the last dose delivery; data were presented as mean ± standard deviation; * *p* < 0.05, ** *p* < 0.01, and *** *p* < 0.001. *p*-trend value was calculated by including the median value of each group as a continuous variable in a general linear model.

As shown in Figure 2A, there were no significant differences in bodyweight among mice in the control and Mn-exposed groups. After six weeks of Mn exposure at different doses, mice triceps Mn concentrations in the 5, 10, and 15 mg/kg Mn-exposed groups were higher than that in the control group, and the average level in the high-dose group was also higher than that in the low-dose group (Figure 2B). However, triceps manganese accumulation did not reach a statistically significant dose–response relationship, which may be due to large individual differences, especially the increase from the 5 mg/kg group to the 10 mg/kg group was not obvious. In addition, forelimb grip strengths of control, 5, 10, and 15 mg/kg Mn-exposed groups were 3.67 ± 0.15, 3.25 ± 0.28, 3.02 ± 0.43, and 2.76 ± 0.55 g/g of bodyweight, respectively. Forelimb grip strengths in 5, 10, and 15 mg/kg Mn-exposed groups were significantly lower as compared to control group (all *p* < 0.05), and a significant dose–response manner was observed (*p*-trend < 0.0001) (Figure 2C).

**Figure 3:**
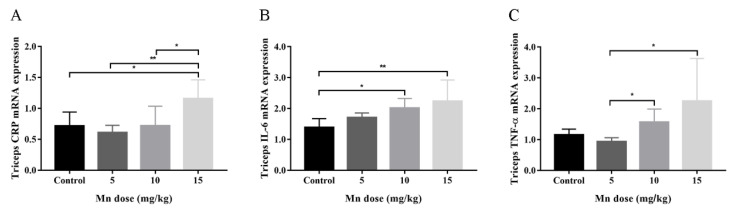
mRNA levels of inflammatory markers and cytokines in control and Mn-exposed mice triceps. (**A**) CRP, (**B**) IL-6, and (**C**) TNF-α. Data were presented as mean ± standard deviation; * *p* < 0.05, ** *p* < 0.01.

The qRT-PCR results showed that CRP mRNA levels of 15 mg/kg Mn-exposed mice triceps were significantly higher than those of control, 5 mg/kg and 10 mg/kg Mn-exposed groups (Figure 3A). IL-6 mRNA levels of 10 mg/kg and 15 mg/kg Mn-exposed mice triceps were significantly higher than that of control (Figure 3B). TNF-α mRNA levels of 10 mg/kg and 15 mg/kg Mn-exposed mice triceps were significantly higher than that of 5 mg/kg Mn-exposed group (Figure 3C).

## 4. Discussion

In the present study, we observed a negative association between urinary Mn concentrations and hand grip strength in elderly men, and this correlation did not change after adjusting for a series of potential confounding factors. Furthermore, our experimental findings demonstrated that Mn exposure significantly reduced forelimb grip strength of male C57BL/6J mice and up-regulated the mRNA expressions of CRP, IL-6, and TNF-α in the triceps muscle. To our knowledge, this is the first study to demonstrate that environmental Mn exposure may serve as a risk factor for diminished grip strength in the elderly, and this association may be related to the inflammatory response in muscle tissue.

Previous studies investigating the relationship between environmental manganese exposure and muscle strength have yielded inconsistent findings. We believe this inconsistency primarily stems from two core factors: differences in exposure assessment methods and variations in the characteristics of study populations. Specifically, the choice of exposure biomarkers, the time windows of exposure they represent, and the underlying susceptibility of the populations are key to explaining these discrepancies. This study opted for creatinine-corrected urinary manganese rather than blood manganese as the exposure marker, a decision based on a comprehensive consideration of their biological characteristics, the target population of this study, and its research objectives. Blood manganese is the most commonly used biomarker for assessing manganese load in the body. However, its short half-life (approximately a few days) means its concentration is susceptible to significant influence from recent dietary intake and metabolic status, thus primarily reflecting short term exposure [43,44,45,46]. In studies of environmental exposure within the general population, blood manganese levels are typically in a low range (e.g., <11 µg/L), and such studies have often failed to find a significant association with grip strength [12]. It is noteworthy, however, that in specific susceptible populations (such as maintenance hemodialysis patients), even blood manganese levels at the high end of the environmental exposure range (>10.6 µg/L) have been associated with an increased risk of sarcopenia [47]. This suggests that certain disease states may modulate their susceptibility to Mn toxicity. In contrast, creatinine-corrected urinary manganese better reflects the body’s manganese metabolic balance and cumulative load over a period, is less affected by short-term fluctuations, and is thus a suitable indicator for assessing long-term, low-dose environmental manganese exposure. Although urinary excretion is a secondary pathway for manganese [48], its detection methods are well-established, reference ranges are clear, and it is widely used in epidemiological studies, facilitating cross-population comparisons [49]. For example, the detection of metal manganese in children near the industrialized area of the southwest coast of Spain used urinary manganese as the standard to verify the level of manganese in hair [50]. This study focuses on community-dwelling elderly men, whose urinary manganese levels (median 0.22 µg/g creatinine) fall within the common environmental exposure range, comparable to levels in general adult males in Northern France [51], higher than those in US males [52], but lower than values reported in some regions of China [53,54,55]. These geographical differences suggest that exposure background and lifestyle factors may influence the body’s manganese burden.

Importantly, even at this environmental exposure level, we still observed a significant negative association and a dose–response relationship between urinary manganese and grip strength. This further indicates that when assessing the potential impact of chronic, non-occupational manganese exposure on muscle function, urinary manganese, which reflects cumulative load, may have higher sensitivity than blood manganese, which reflects immediate concentration. This is particularly relevant for revealing the effects of low-level, long-term exposure associated with progressive muscle strength decline in the general elderly population.

Prior studies also primarily targeted populations with occupational Mn poisoning (where severe motor dysfunction was observed [56]) or children/adolescents (where no association between Mn and grip strength was reported [13]), while our study is the first to focus on the general elderly population, a group not previously targeted in this context. By detecting an association between environmental-level Mn exposure and grip strength in this demographic, we extend current knowledge of Mn’s “low-dose toxicity” and provide novel evidence for the early health effects of Mn exposure. Additionally, previous studies on manganese poisoning in occupational populations have shown that manganese exposure may impair motor function such as muscle weakness [57,58,59]. This provides indirect support for the negative association between Mn content and grip strength observed in the present study. Animal research on Mn and grip strength similarly remains limited and inconsistent: Krishna et al. (2014) observed decreased forelimb grip strength in adult male C57BL/6 mice after 6 weeks of 0.4 g/L Mn exposure through drinking water [15]. Nkpaa et al. (2021) also observed decreased forelimb grip strength in adult male Wistar rats after 35 days of 30 mg/kg Mn exposure by oral gavage [16]. But when Dodd CA et al. (2005) gave male C57BL/6 retired breeder mice a single subcutaneous injection of either 0, 50, or 100 mg/kg of MnCl2 or three injections of either of these doses over 7 days, no significant main effect of dose level on grip strength or grip fatigue was observed [14]. The differences in these results may be caused by factors including exposure routes, Mn doses, exposure time, animal species, animal age, and grip strength measurement methods. Thus, in order to validate our epidemiological result and the discrepancy of previous animal studies, we further conducted an animal experiment. Different from the aforementioned studies, we chose an intraperitoneal injection for Mn exposure in mice. Our dosages of Mn exposure were set according to previous studies [60,61]. Using an animal model with a more direct route of Mn exposure and a subacute exposure time (6 weeks), we verified that Mn exposure could decrease forelimb grip strength in mice. Collectively, our epidemiological and animal data support a dose–response relationship between Mn exposure and grip strength impairment across species.

Our animal experiments further showed that Mn exposure up-regulated the mRNA expression of CRP, IL-6 and TNF-α in triceps muscle of mice, suggesting that inflammatory response may play an important role in the decline of grip strength induced by manganese exposure. In recent years, skeletal muscle has been recognized as an organ producing and releasing cytokines named as myokines [62], and IL-6 is one of the myokines that can induce muscle wasting. In non-Mn contexts, such as sarcopenia or cancer cachexia, it activates the JAK-STAT3 signaling pathway, which subsequently upregulates muscle-specific ubiquitin ligases, including Atrogin-1 and MuRF1. These ligases are key mediators of muscle protein degradation [63]. TNF-α, another well-established driver of muscle wasting, exerts direct catabolic effects by activating the NF-κB pathway, which promotes proteolysis via the ubiquitin-proteasome system and inhibits muscle protein synthesis [64,65]. In an aged mouse model of sarcopenia, higher TNF-α levels were associated with a faster rate of decline in grip strength [66], which is consistent with our observation that “manganese-induced up-regulation of TNF-α is accompanied by decline in grip strength”. CRP is an acute phase reactant protein that is mainly produced and secreted into circulation by the liver during infection, inflammation, and tissue damage [67]. Locally produced CRP within tissues could exert local or systemic effects through blood flow, and the expression of CRP could be regulated by cytokines such as IL-6 and TNF-α [68]. Notably, in the general population, higher circulating CRP, IL-6, and TNF-α levels are associated with lower grip strength [34], and occupational Mn exposure increases serum levels of these markers and cytokines [69]. By linking Mn to this well-characterized inflammatory pathway, our study places Mn-induced grip strength reduction within a broader framework of inflammatory muscle dysfunction, enhancing its biological plausibility.

Against the backdrop of a rapidly aging global population, identifying modifiable risk factors for grip strength decline is critical for promoting healthy aging, and our findings carry three key public health implications. First, the stronger association between Mn-grip strength in elderly men aged 66 years and older, as well as those with a BMI below 23.9 kg/m^2^, suggests that these subgroups may benefit from targeted interventions, such as monitoring Mn exposure and providing dietary counseling to maintain a healthy BMI [70]. Second, smoking is both a source of Mn exposure (via tobacco-derived Mn [71]) and an independent risk factor for grip strength decline [72,73,74], and our data show it masks the Mn-grip strength association. Reducing smoking could simultaneously lower Mn exposure and directly protect muscle function, making it a high-impact intervention.

This study possessed several strengths. To the best of our knowledge, this is the first effort to explore the relationship between Mn exposure and grip strength in the elderly population. Extensive data on a series of important potential confounding factors, such as age, education level, occupation, BMI, living habits and physical conditions, were collected and controlled in the sequential models. Moreover, we also adjusted for Cd, which had been reported to have an inverse association with grip strength [28,29,75], so that the influence of possible confounder could be further reduced. More importantly, the weakening effect of Mn on grip strength was also validated in an animal study in the present study, and a possible mechanism was proposed as well. Nevertheless, a few limitations are also noteworthy. First of all is the cross-sectional design of our epidemiological study, which precludes temporality of the association observed, thus we cannot entirely establish a causal relationship between Mn exposure and grip strength. Second, a single measurement of Mn concentrations with first morning urine samples of elderly men is still not enough to completely reflect intra-individual variability and long-term average exposure. Third, we only recruited elderly men and the sample size was relatively small, thus precaution has to be taken in extending the results to elderly women. Fourth, the residual confounding from dietary, which is the main exposure route of Mn, has not been ruled out, even though a number of other confounders were controlled. Fifth, in our animal study, there was an age discrepancy between the initial exposure age of the mice (6 weeks old) and the elderly male population in the epidemiological study (with an average age of 66 years). In addition, the exposure duration was only six weeks, which did not adequately model the effects of long-term environmental Mn exposure. At the same time, it must be acknowledged that the manganese doses administered via intraperitoneal injection in this toxicological study are at levels that significantly exceed typical human environmental exposure. Although this design is valuable for clarifying potential mechanisms such as inflammation, it limits the direct correlation between the experimental results and the environment. Future research will need to use models that are closer to environmental exposure (e.g., chronic drinking water exposure) to further confirm these findings. Finally, we only detected the mRNA levels of inflammatory factors and did not assess their protein expression or activity. Future research should verify the above results at the protein level and deeply explore related downstream pathways (such as JAK-STAT3, NF-ĸB), and further systematically clarify the inflammatory regulatory mechanisms in manganese-related muscle dysfunction through intervention studies (such as gene knockout models or anti-inflammatory treatments).

## 5. Conclusions

In summary, we found a significant inverse correlation between urinary Mn concentrations and grip strength in the elderly men, and the association remained robust after sequential adjustments for confounders. Additionally, the inverse association between Mn exposure and grip strength was further validated in male C57BL/6J mice. Given that lower grip strength in the elderly is related to higher risks of morbidity and mortality, which affects life quality and well-being of the aging population, the present results may have substantive public health implications for the elderly. Subsequent epidemiologic studies, especially longitudinal designs with a larger sample, are warranted to further confirm the association observed in the current study and clarify the potential causation of Mn exposure with the decline of grip strength.

## Figures and Tables

**Table 1:** Baseline Characteristics, Urinary Mn Levels and Hand Grip Strength of the Study Population (*n* = 375).

Characteristics	Mean ± SD or No. (%)	Mn (μg/g Creatinine) ^a^	*p*-Value	Hand Grip Strength (kg) ^a^	*p*-Value
Overall	375 (100.00)	0.22 (0.14, 0.42)		28.1 (22.3, 33.6)	
Age (years)	66.03 ± 3.92		0.529		<0.0001
60–64	156 (41.60)	0.23 (0.13, 0.41)		29.8 (24.7, 35.2)	
65–69	138 (36.80)	0.22 (0.14, 0.40)		28.2 (22.3, 32.3)	
≥70	81 (21.60)	0.23 (0.16, 0.49)		25.2 (20.5, 29.3)	
Ethnicity			0.342		0.636
* Han*	28 (7.47)	0.30 (0.14, 0.71)		28.2 (24.8, 35.0)	
* Zhuang*	340 (90.67)	0.22 (0.14, 0.39)		28.2 (22.3, 33.6)	
Other	7 (1.87)	0.24 (0.19, 0.38)		23.0 (22.0, 33.3)	
Marital status			0.032		0.065
Married	331 (88.27)	0.22 (0.14, 0.37)		28.4 (22.5, 33.9)	
Other	44 (11.73)	0.36 (0.14, 0.71)		26.6 (20.6, 31.8)	
Education level			0.194		<0.0001
No formal education	36 (9.60)	0.27 (0.17, 0.36)		27.6 (21.0, 30.2)	
1–6 years of education	155 (41.33)	0.22 (0.15, 0.43)		26.5 (20.8, 31.6)	
>6 years of education	184 (49.07)	0.21 (0.12, 0.40)		29.8 (24.6, 35.0)	
Occupation			0.029		0.708
Farmer	316 (84.27)	0.23 (0.14, 0.44)		28.1 (22.5, 33.5)	
Non-farmer	59 (15.73)	0.19 (0.09, 0.31)		27.7 (21.6, 34.5)	
Household income (RMB/year)			0.604		0.391
<10,000	147 (39.20)	0.23 (0.14, 0.42)		28.3 (22.5, 33.7)	
10,000–30,000	82 (21.87)	0.24 (0.13, 0.49)		27.5 (21.8, 31.9)	
≥30,000	146 (38.93)	0.21 (0.14, 0.37)		28.6 (22.4, 34.2)	
BMI (kg/m^2^)	21.69 ± 2.86		0.098		0.010
<18.5	44 (11.73)	0.20 (0.13, 0.28)		25.5 (20.0, 29.8)	
18.5–23.9	257 (68.53)	0.23 (0.14, 0.48)		28.0 (22.3, 33.4)	
≥24.0	74 (19.73)	0.21 (0.13, 0.37)		30.1 (24.6, 34.8)	
Tobacco smoking			0.902		0.635
No	199 (53.07)	0.22 (0.14, 0.43)		28.0 (22.3, 33.2)	
Yes	176 (46.93)	0.23 (0.14, 0.39)		28.2 (22.4, 33.9)	
Alcohol drinking			0.212		0.235
No	168 (44.80)	0.21 (0.13, 0.39)		28.8 (22.9, 33.7)	
Yes	207 (55.20)	0.24 (0.14, 0.42)		27.6 (21.9, 33.3)	
Farm-work			0.144		0.240
No	107 (28.53)	0.21 (0.11, 0.37)		27.9 (22.3, 32.0)	
Yes	268 (71.47)	0.23 (0.14, 0.44)		28.2 (22.3, 34.2)	
Hypertension			0.529		0.879
No	140 (37.33)	0.23 (0.14, 0.39)		28.0 (22.2, 33.7)	
Yes	235 (62.67)	0.22 (0.13, 0.42)		28.2 (22.4, 33.5)	

Abbreviations: SD, standard deviation; No., number; BMI, body mass index. a Data are presented as median (interquartile range).

**Table 2:** *β* (95% CI) values in hand grip strength (kg) by urinary Mn concentrations using general linear model (*n* = 375).

Urinary Mn (μg/g Creatinine)	Model A *^a^*	Model B *^b^*	Model C *^c^*	Model D *^d^*
	*β* (95%CI)	*β* (95%CI)	*β* (95%CI)	*β* (95%CI)
T_1_ (<0.17)	Reference	Reference	Reference	Reference
T_2_ (0.17–0.33)	−3.60 (−5.71, −1.48)	−3.36 (−5.42, −1.30)	−3.41 (−5.46, −1.37)	−3.33 (−5.39, −1.27)
T_3_ (≥0.33)	−3.91 (−6.02, −1.79)	−3.58 (−5.65, −1.51)	−3.73 (−5.79, −1.67)	−3.57 (−5.68, −1.47)
*p*-trend	0.003	0.006	0.003	0.007

Abbreviations: CI, confidence interval; T, tertile. *^a^* crude model. *^b^* adjusted for age (60–64, 65–69, ≥70 years), ethnicity (Han, Zhuang, other), marital status (married, other), education level (no formal education, 1–6 years of education, >6 years of education), and occupation (farmer, non-farmer). *^c^* adjusted for Model B covariates plus BMI (<18.5, 18.5–23.9, ≥24.0 kg/m^2^), smoking (yes, no), drinking (yes, no), farm-work (yes, no), and hypertension (yes, no). *^d^* adjusted for Model C covariates plus urinary Cd concentrations.

## Data Availability

Data is contained within the article or Supplementary Material.

## Supplementary Materials

The following supporting information can be downloaded at: https://www.mdpi.com/article/10.3390/toxics14010031/s1. Table S1. Primer sequences used for qRT-PCR. Table S2. Association between urinary Mn levels and hand grip strength, stratified by median age, BMI, and smoking status. Table S3. Composition of the Rat & Mouse Maintenance Diet Used in This Study. Table S4. Distributions of urinary Cd concentrations according to baseline characteristics.
