# Supplementary material for: Manganese Exposure Is Associated with Reduced Grip Strength: Evidence from Humans and Mice
Source: Toxics. 2025 Dec 26;14(1):31. doi: 10.3390/toxics14010031 (PMC12846208; doi:10.3390/toxics14010031)
Supplement: Supplementary file 1 [file toxics-14-00031-s001.zip › toxics-4023204-supplementary.pdf]

Supplementary Material for:

Manganese Exposure Is Associated with Reduced Grip Strength: Evidence from Humans and Mice

Peiyu Fang [1,2,†], Chuanqiao Tang [1,2,†], Shengtao Wei [1,2], Wenmin Lu [1,2], Shaohui Liu [1,2], Xiaoli Ma [1,2], Li'e Zhang [2,3], Guiqiang Liang [1,2], Jian Wang [2,3], Yang Peng [2,3,*] and Yunfeng Zou [1,2,*]

[1] Department of Toxicology, School of Public Health, Guangxi Medical University, Nanning 530021, China; fangpeiyu@sr.gxmu.edu.cn (P.F.); clearpast@163.com (C.T.); weishengtao@stu.gxmu.edu.cn (S.W.); luwenmin@stu.gxmu.edu.cn (W.L.); liushaohui@stu.gxmu.edu.cn (S.L.); maxiaoli@sr.gxmu.edu.cn (X.M.); liangguiqiang@gxmu.edu.cn (G.L.)

[2] Guangxi Key Laboratory of Environment and Health Research, Guangxi Medical University,
Nanning 530021, China; zhang-121704151010@163.com (L.Z.); wangjian@gxmu.edu.cn (J.W.)

[3] Department of Occupational and Environmental Health, School of Public Health, Guangxi Medical
University, Nanning 530021, China

* Correspondence: pyu098@126.com (Y.P.); zouyunfeng@gxmu.edu.cn (Y.Z.); Tel.: +86-771-5358269 (Y.Z.)

† These authors contributed equally to this work.

Table of contents:

Table S1. Primer sequences used for qRT-PCR

Table S2. Association between urinary Mn levels and hand grip strength, stratified by median age, BMI, and smoking status

Table S3. Composition of the Rat & Mouse Maintenance Diet Used in This Study

Table S4. Distributions of urinary Cd concentrations according to baseline characteristics.

Table S1. Primer Sequences Used for qRT-PCR

Gene	Forward primer	Reverse primer
CRP	5'-CCTGAGGCTCCAACACACAT-3'	5'-GTGTAGCCCTTGTGCAGACT-3'
IL-6	5'-CCAATTTCCAATGCTCTCC-3'	5'-ACCACAGTGAGGAATGTCCA-3'
TNF-α	5'-CTGAACTTCGGGGTGATCGG-3'	5'-GGTTTGTCACTCGAATTTTGAGA-3'
GAPDH	5'-CCTTCCGTGTTCCTACCCC-3'	5'-GCCCAAGATGCCCTTCAGT-3'

Table S2. Association between Urinary Mn Levels and Hand Grip Strength, Stratified by Age, BMI, and Smoking Status

**Urinary Mn (μg/g creatinine)**	***β* (95% CI)** [a]	***P*-interaction**
Age (years)		0.649
< 66 (*n* = 180)		
T_1 (< 0.17)	Reference	
T_2 (0.17-0.36)	-2.90 (-6.17, 0.37)	
T_3 (≥ 0.36)	-2.25 (-5.43, 0.93)	
P-trend	0.311	
≥ 66 (*n* = 195)		
T_1 (< 0.17)	Reference	
T_2 (0.17-0.30)	**-3.28 (-5.94, -0.63)**	
T_3 (≥ 0.30)	**-5.37 (-8.16, -2.58)**	
P-trend	**0.001**	
BMI (kg/m^2)		0.851
< 18.5 (*n* = 44)		
T_1 (< 0.16)	Reference	
T_2 (0.16-0.23)	**-7.40 (-12.95, -1.85)**	
T_3 (≥ 0.23)	-5.45 (-11.85, 0.95)	
P-trend	0.214	
18.5-23.9 (*n* = 257)		

T1 (< 0.17)	Reference	
T2 (0.17-0.36)	**-3.37 (-5.77, -0.96)**	
T3 (≥ 0.36)	**-3.33 (-5.67, -0.98)**	
P-trend	**0.034**	
≥ 24.0 (*n* = 74)		
T1 (< 0.16)	Reference	
T2 (0.16-0.30)	-4.54 (-11.12, 2.04)	
T3 (≥ 0.30)	-6.60 (-13.69, 0.49)	
P-trend	0.092	
Tobacco smoking		**0.025**
No (*n* = 199)		
T1 (< 0.16)	Reference	
T2 (0.16-0.33)	**-4.55 (-7.43, -1.67)**	
T3 (≥ 0.33)	**-6.10 (-9.01, -3.19)**	
P-trend	**<0.001**	
Yes (*n* = 176)		
T1 (< 0.17)	Reference	
T2 (0.17-0.33)	-1.98 (-5.01, 1.04)	
T3 (≥ 0.33)	-0.93 (-4.07, 2.22)	
P-trend	0.849	

Abbreviations: CI, confidence interval; T, tertile. BMI, body mass index.

[a] These models were adjusted for covariates except for the corresponding

stratified factors. Covariates included age (60-64, 65-69, ≥ 70 years), ethnicity (*Han*, *Zhuang*, else), marital status (married, else), education level (no formal education, 1-6 years of education, > 6 years of education), occupation (farmer, non-farmer), BMI (< 18.5, 18.5-23.9, ≥ 24.0 kg/m^2), smoking (yes, no), drinking (yes, no), farm-work (yes, no), hypertension (yes, no), and urinary Cd concentrations.

Table S3. Composition of the Rat & Mouse Maintenance Diet Used in This Study

Item Category	Item	Content / Specification
**Product name**	Rat & Mouse Maintenance Diet	Keao Xieli Feed (KeaoxieliFeed®)
**Physical form**	Pellet shape & size	Cylindrical pellet, 12 mm diameter
**Packaging**	Regular pack / SPF pack	25 kg/bag; SPF vacuum 10 kg/carton
**Feeding regimen**	Ad libitum access	Fresh water simultaneously available
**Nutritional Indicator**	Moisture	≤10%
	Crude Fat	≥4%
	Crude Ash	≤8%
	Phosphorus	0.6 - 1.2%
	Crude Protein	≥18%
	Crude Fiber	≤5%
	Calcium	1.0 - 1.8%
	Ash	≤ 8 %
**Minerals**	Sodium	3.1 g kg^{-1}
	Magnesium	2.9 g kg^{-1}
	Potassium	7.4 g kg^{-1}
	Copper	11.4 mg kg^{-1}
	Iron	113.7 mg kg^{-1}
	Manganese	80 mg kg^{-1}
	Zinc	31.6 mg kg^{-1}
	Selenium	0.20 mg kg^{-1}
	Iodine	0.70 mg kg^{-1}
**Amino acids**	Methionine + Cystine	5.80 g kg^{-1}
	Lysine	8.90 g kg^{-1}
	Tryptophan	2.10 g kg^{-1}
	Arginine	9.90 g kg^{-1}
	Leucine	14.80 g kg^{-1}
	Isoleucine	7.40 g kg^{-1}
	Threonine	6.60 g kg^{-1}
	Valine	8.90 g kg^{-1}
	Histidine	4.90 g kg^{-1}
	Benzene + tyrosine	14.60 g kg^{-1}

Item Category	Item	Content / Specification
**Vitamins**	Vitamin A	7 800 IU kg^{-1}
	Vitamin D_3	1 200 IU kg^{-1}
	Vitamin E	67 mg kg^{-1}
	Vitamin K	5 mg kg^{-1}
	Vitamin B_1	10 mg kg^{-1}
	Vitamin B_2	15 mg kg^{-1}
	Vitamin B_6	10 mg kg^{-1}
	Vitamin B_{12}	0.02 mg kg^{-1}
	Niacin	55 mg kg^{-1}
	Pantothenic acid	22 mg kg^{-1}
	Biotin	0.20 mg kg^{-1}
	Folic acid	6.6 mg kg^{-1}
	Choline	1 250 mg kg^{-1}
**Calculated energy**	Protein	23.07%
	Fat	11.85%
	Carbohydrate	65.08%
**Gross energy**		3.40 kcal g^{-1} (≈14.2 kJ g^{-1})

Table S4. Distributions of urinary Cd concentrations according to baseline characteristics.

Characteristic	N	Uncorrected Cd concentration (μg/L)		Creatinine-corrected Cd concentration (μg/g creatinine)	
		GM (95% CI)	*p*	GM (95% CI)	*p*
**All**	375	1.56 (1.43, 1.70)		1.59 (1.48, 1.71)	
**Age (years)**			0.829		0.837
60-64	156	1.50 (1.30, 1.73)		1.58 (1.40, 1.78)	
65-69	138	1.63 (1.42, 1.87)		1.58 (1.40, 1.78)	
≥ 70	81	1.56 (1.32, 1.84)		1.64 (1.44, 1.88)	
**BMI (kg/m^2)**			**0.011**		**0.016**
< 18.5	44	1.88 (1.48, 2.38)		1.67 (1.36, 2.05)	
18.5-23.9	257	1.61 (1.45, 1.79)		1.68 (1.54, 1.83)	
≥ 24	74	1.23 (1.04, 1.47)		1.29 (1.09, 1.51)	
**Education level**			0.773		0.623
No formal educated	36	1.80 (1.38, 2.34)		1.77 (1.37, 2.28)	
1-6 years of education	155	1.51 (1.32, 1.74)		1.62 (1.45, 1.81)	
7 or more years of education	184	1.55 (1.37, 1.75)		1.53 (1.38, 1.70)	
**Occupation**			0.133		0.375
Farmer	316	1.60 (1.46, 1.75)		1.61 (1.49, 1.74)	
Non-farmer	59	1.35 (1.07, 1.70)		1.48 (1.22, 1.78)	
**Household income (RMB/year)**			0.110		**0.028**
< 10000	147	1.59 (1.38, 1.82)		1.69 (1.51, 1.88)	
10000-30000	82	1.78 (1.49, 2.13)		1.79 (1.53, 2.10)	
> 30000	146	1.42 (1.24, 1.63)		1.40 (1.24, 1.58)	
**Marital status**			0.412		0.075
Married	331	1.54 (1.40, 1.69)		1.55 (1.44, 1.68)	
Single/divorced/widowed	44	1.70 (1.35, 2.15)		1.89 (1.59, 2.24)	
**Tobacco Smoking**			**< 0.001**		**< 0.001**
Yes	176	2.04 (1.80, 2.30)		2.00 (1.82, 2.21)	
No	199	1.23 (1.10, 1.37)		1.30 (1.18, 1.43)	
**Alcohol Drinking**			0.719		0.145
Yes	207	1.52 (1.34, 1.71)		1.50 (1.36, 1.66)	
No	168	1.61 (1.43, 1.81)		1.70 (1.53, 1.90)	

Abbreviations: GM, geometric mean; CI, confidence interval; BMI, body mass

index.
